# Supplementary material for: Effectiveness of CKD Exacerbation Countermeasures in Izumo City
Source: J Pers Med. 2021 Oct 28;11(11):1104. doi: 10.3390/jpm11111104 (PMC8622121; doi:10.3390/jpm11111104)
Supplement: Supplementary file 1 [file jpm-11-01104-s001.zip › jpm-1406755-supplementary.pdf]

Supplementary Table S1. Baseline characteristics of adults with specific health checkups in FY2018 and FY2019

	FY2018	FY2019
Number*	11,061	10,717
Age, years	68 [64–71]	69 [64–72]
Male, n (%)	4,969 (44.9)	4,833 (45.1)
Current smokers, n (%)	1,334 (12.1)	1,298 (12.1)
Drinking frequency, Rare, n (%)	5,780 (52.3)	5,617 (52.4)
Occasional	2,231 (20.2)	2,150 (20.1)
Daily	3,050 (27.6)	2,950 (27.5)
Body mass index, kg/m^2	23.0 ± 3.4	22.9 ± 3.5
Systolic blood pressure, mmHg	132 ± 17	131 ± 17
Diastolic blood pressure, mmHg	77 ± 11	77 ± 11
Triglyceride, mg/dL	97 [70–137]	96 [68–137]
Low-density lipoprotein cholesterol, mg/dL	121 ± 30	119 ± 30
Hemoglobin A1c, %	5.7 ± 0.6	5.8 ± 0.7
eGFR, $mL/min/1.73m^2$	75 ± 15	73 ± 14
Urinary protein, negative, n (%)	9,277 (83.9)	8,946 (83.5)
±	1,061 (9.6)	1,040 (9.7)
1+	512 (4.6)	507 (4.7)
≥2+	211 (1.9)	224 (2.1)
Current treatment for hypertension, n (%)	4,358 (39.4)	4,293 (40.1)
dyslipidemia	3,095 (28.0)	3,069 (28.6)
diabetes	925 (8.4)	890 (8.3)
History of cardiovascular disease, n (%)	873 (7.9)	883 (8.2)

Data are presented as mean ± standard deviation, median (25%–75%), or n (%)

eGFR, estimated glomerular filtration rate

* Adults who had no missing baseline data at specific health checkups

Supplementary Table S2. Baseline characteristics of CKD re-examination candidates in FY2018 and FY2019

	FY2018	FY2019	P-value
Number*	826	864	
Age, years	69 [66–72]	70 [66–72]	0.840
Male, n (%)	630 (76.3)	677 (78.4)	0.306
Current smokers, n (%)	120 (14.5)	142 (16.4)	0.279
Drinking frequency, Rare, n (%)	372 (45.0)	417 (48.3)	0.117
Occasional	167 (20.2)	142 (16.4)	
Daily	287 (34.7)	305 (35.3)	
Body mass index, kg/m^2	24.0 ± 3.6	24.2 ± 3.5	0.289
Systolic blood pressure, mmHg	134 ± 18	133 ± 18	0.348
Diastolic blood pressure, mmHg	79 ± 12	79 ± 12	0.739
Triglyceride, mg/dL	116 [81–172]	117 [83–166]	0.927
Low-density lipoprotein cholesterol, mg/dL	116 ± 33	113 ± 31	0.083
Hemoglobin A1c, %	5.9 ± 0.8	6.0 ± 0.9	0.110
eGFR, mL/min/1.73m^2	53 ± 14	54 ± 13	0.600
Urinary protein, negative, n (%)	478 (57.9)	527 (61.0)	0.416
±	84 (10.2)	70 (8.1)	
1+	60 (7.3)	62 (7.2)	
≥2+	204 (24.7)	205 (23.7)	
Current treatment for hypertension, n (%)	510 (61.7)	514 (59.5)	0.343
dyslipidemia	277 (33.5)	308 (35.6)	0.361
diabetes	137 (16.6)	149 (17.2)	0.718
History of cardiovascular disease, n (%)	114 (13.8)	134 (15.5)	0.321

Data are presented as mean ± standard deviation, median (25%–75%), or n (%)

eGFR, estimated glomerular filtration rate

* CKD re-examination candidates who had no missing baseline data at specific health checkups

Supplementary Table S3. Baseline characteristics of CKD re-examination implementation in FY2018 and FY2019

	FY2018	FY2019	P-value
Number*	826	864	
Age, years	69 [66–72]	70 [66–72]	0.840
Male, n (%)	630 (76.3)	677 (78.4)	0.306
Current smokers, n (%)	120 (14.5)	142 (16.4)	0.279
Drinking frequency, Rare, n (%)	372 (45.0)	417 (48.3)	0.117
Occasional	167 (20.2)	142 (16.4)	
Daily	287 (34.7)	305 (35.3)	
Body mass index, kg/m^2	24.0 ± 3.6	24.2 ± 3.5	0.289
Systolic blood pressure, mmHg	134 ± 18	133 ± 18	0.348
Diastolic blood pressure, mmHg	79 ± 12	79 ± 12	0.739
Triglyceride, mg/dL	116 [81–172]	117 [83–166]	0.927
Low-density lipoprotein cholesterol, mg/dL	116 ± 33	113 ± 31	0.083
Hemoglobin A1c, %	5.9 ± 0.8	6.0 ± 0.9	0.110
eGFR, mL/min/1.73m^2	53 ± 14	54 ± 13	0.600
Urinary protein, negative, n (%)	478 (57.9)	527 (61.0)	0.416
±	84 (10.2)	70 (8.1)	
1+	60 (7.3)	62 (7.2)	
≥2+	204 (24.7)	205 (23.7)	
Current treatment for hypertension, n (%)	510 (61.7)	514 (59.5)	0.343
dyslipidemia	277 (33.5)	308 (35.6)	0.361
diabetes	137 (16.6)	149 (17.2)	0.718
History of cardiovascular disease, n (%)	114 (13.8)	134 (15.5)	0.321

Data are presented as mean ± standard deviation, median (25%–75%), or n (%)

eGFR, estimated glomerular filtration rate

* Adults who underwent CKD re-examination and had no missing baseline data at specific health checkups
